# Impact of randomised *w*mel *Wolbachia* deployments on notified dengue cases and insecticide fogging for dengue control in Yogyakarta City

**DOI:** 10.1080/16549716.2023.2166650

**Published:** 2023-01-26

**Authors:** Citra Indriani, Stephanie K. Tanamas, Uswatun Khasanah, Muhammad Ridwan Ansari, Rubangi, Warsito Tantowijoyo, Riris Andono Ahmad, Suzanne M. Dufault, Nicholas P. Jewell, Adi Utarini, Cameron P. Simmons, Katherine L. Anders

**Affiliations:** a World Mosquito Program Yogyakarta, Centre for Tropical Medicine, Faculty of Medicine, Public Health and Nursing, Universitas Gadjah Mada, Yogyakarta, Indonesia; b Department of Biostatistics, Epidemiology and Population Health, Faculty of Medicine Public Health and Nursing, Universitas Gadjah Mada, Yogyakarta, Indonesia; c World Mosquito Program, Monash University, Clayton, VIC, Australia; d Disease Control Department, Yogyakarta City Health Office, Yogyakarta, Indonesia; e Division of Pulmonary and Critical Care Medicine, School of Medicine, University of California, San Francisco, CA, USA; f Department of Medical Statistics, London School of Hygiene and Tropical Medicine, London, UK; g Department of Health Policy and Management, Faculty of Medicine Public Health and Nursing, Universitas Gadjah Mada, Yogyakarta, Indonesia

**Keywords:** Dengue, *Wolbachia*, *w*Mel, *Aedes aegypti*, vector control

## Abstract

**Background:**

Releases of Wolbachia (*w*Mel)-infected Aedes aegypti mosquitoes significantly reduced the incidence of virologically confirmed dengue in a previous cluster randomised trial in Yogyakarta City, Indonesia. Following the trial, *w*Mel releases were extended to the untreated control areas, to achieve city-wide coverage of Wolbachia.

**Objective:**

In this predefined analysis, we evaluated the impact of the wMel deployments in Yogyakarta on dengue hemorrhagic fever (DHF) case notifications and on the frequency of perifocal insecticide spraying by public health teams.

**Methods:**

Monthly counts of DHF cases notified to the Yogyakarta District Health Office between January 2006 and May 2022 were modelled as a function of time-varying local wMel treatment status (fully- and partially-treated vs untreated, and by quintile of wMel prevalence). The frequency of insecticide fogging in wMel-treated and untreated areas was analysed using negative binomial regression.

**Results:**

Notified DHF incidence was 83% lower in fully treated vs untreated periods (IRR 0.17 [95% CI 0.14, 0.20]), and 78% lower in areas with 80–100% wMel prevalence compared to areas with 0–20% *w*Mel (IRR 0.23 [0.17, 0.30]). A similar intervention effect was observed at 60–80% wMel prevalence as at 80–100% prevalence (76% vs 78% efficacy, respectively). Pre-intervention, insecticide fogging occurred at similar frequencies in areas later randomised to wMel-treated and untreated arms of the trial. After *w*Mel deployment, fogging occurred significantly less frequently in treated areas (IRR 0.17 [0.10, 0.30]).

**Conclusions:**

Deployments of *w*Mel-infected Aedes aegypti mosquitoes resulted in an 83% reduction in the application of perifocal insecticide spraying, consistent with lower dengue case notifications in wMel-treated areas. These results show that the Wolbachia intervention effect demonstrated previously in a cluster randomised trial was also measurable from routine surveillance data.

## Introduction

Dengue is the most prevalent mosquito-borne viral disease worldwide, with an estimated 4 billion people living in tropical and subtropical areas of 129 countries currently at risk of infection [1]. The *Aedes aegypti* mosquito transmits the four serotypes of dengue virus (DENV) between humans, and thrives in urban environments where domestic water-holding containers provide plentiful larval habitats in close proximity to human blood meals. Dengue control programs have traditionally focused on reducing vector abundance via environmental management, larval control and insecticide spraying, and on reducing human-mosquito contact with personal protection measures. However, the cost and resources required to sustain vector control activities at scale, and the evolution and spread of insecticide resistance, have limited the effectiveness of this approach [2]. The increasing occurrence of explosive dengue outbreaks in tropical cities and previously unaffected populations, together with the emergence of pandemic transmission of the *Aedes*-borne chikungunya and Zika viruses in the mid-2010s, highlights the need for better tools [3]. In 2022, the World Health Organization launched a Global Integrated Arbovirus Initiative to address the growing threat of *Aedes*-borne viruses to global health [4].

An emerging evidence-based method for dengue control harnesses the virus-blocking properties of some strains of *Wolbachia pipientis*, a maternally inherited, obligate intracellular bacteria that is common in many insect species, including mosquitoes, but which does not naturally occur in *Ae. aegypti* mosquitoes [5,6]. Short-term field releases of *Ae. aegypti* infected with the *w*Mel strain of *Wolbachia* (derived from *Drosophila melanogaster*) in communities in Australia, Asia and South America have resulted in sustained introgression of *w*Mel into local *Ae. aegypti* populations, with a subsequent reduction in the incidence of dengue, chikungunya and Zika [7–10]. The efficacy of the *Wolbachia* introgression method was demonstrated in a cluster randomised controlled trial conducted in Yogyakarta, Indonesia, in 2018 – 2020 (the Applying *Wolbachia* to Eliminate Dengue [AWED] trial [11]), in which patients with acute febrile illness presenting to a network of primary care clinics were prospectively enrolled and tested for dengue virus (DENV) infection. The primary analysis of the AWED trial demonstrated that the incidence of virologically-confirmed dengue (VCD) cases was reduced by 77%, and hospitalised VCD cases by 86%, in *w*Mel-treated clusters compared to untreated areas [12].

National passive disease surveillance systems are set up to detect outbreaks of disease and inform public health response. Passive disease surveillance systems capture general trends and local signals of disease activity [13], but are less reliable for accurately quantifying disease burden or for determining the effectiveness of public health interventions. Surveillance data tend to underestimate disease burden resulting from a combination of under-ascertainment of cases, incomplete reporting, and inadequate resources for diagnostic confirmation. In Indonesia, the dengue surveillance system is thought to underestimate the ‘true’ dengue burden by a factor of 11.5 [14], with other estimates ranging from a factor of 5 [15] to as high as 126 [16]. Nevertheless, surveillance systems provide readily accessible data often collected over long time periods, and can be a valuable, pragmatic alternative data source to randomised controlled trials (RCT) and prospective observational studies where these are not possible due to time, financial or other logistical constraints. The interrupted time-series (ITS) approach is a quasi-experimental method which utilises a series of repeated measures taken at regular time intervals to establish a baseline trend, and which has been used to assess the effectiveness of a range of different public health interventions [17,18]. Previous studies have determined that RCT results can be reproduced using ITS under the right conditions [19–24].

The purpose of the current study is two-fold. First, to determine whether the efficacy of *w*Mel deployments reported in the primary analysis of the AWED trial was also measurable by interrupted time series analysis of hospitalised dengue haemorrhagic fever (DHF) cases notified to the routine disease surveillance system. Second, to examine whether reduced dengue incidence resulting from the *w*Mel intervention was reflected in a change in the frequency of reactive perifocal insecticide spraying by public health teams.

## Methods

### Study design

**Figure 1.**
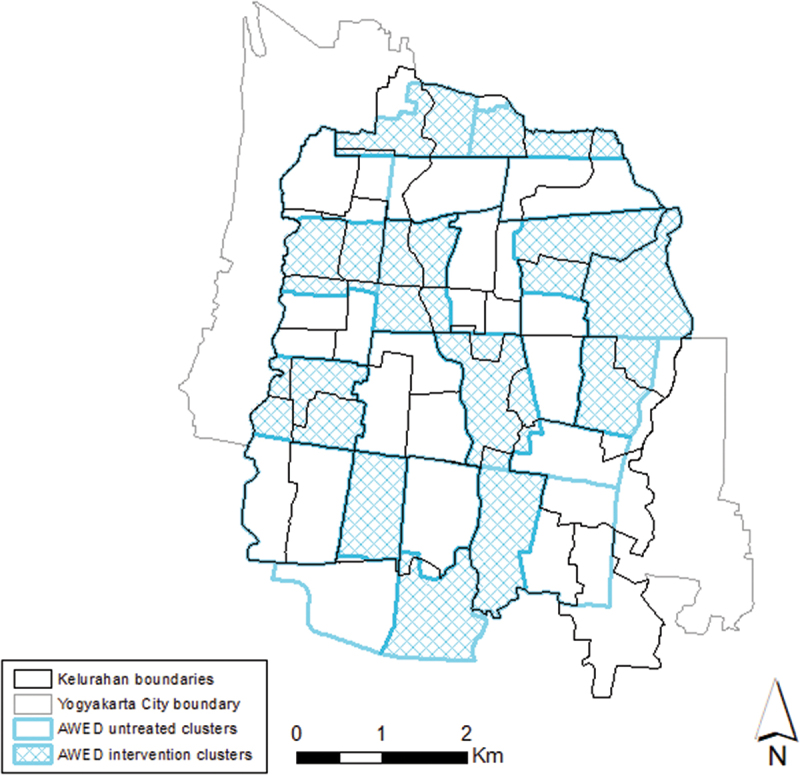
Map of Yogyakarta City (city boundary in grey) showing the boundaries of the 35 kelurahans (black lines) within the AWED (Applying *Wolbachia* to Eliminate Dengue) trial site overlaid on the cluster boundaries (blue lines) used to define *w*mel intervention clusters (blue shading) and untreated clusters (hollow). The population of the 35 kelurahans was approximately 313,000 in 2017.

The AWED cluster randomised trial site in Yogyakarta City, Indonesia, encompassed 35 administrative divisions called kelurahans (urban villages) with a population in 2017 of approximately 313,000 (Figure 1). The study site was divided into 24 contiguous clusters each approximately 1 km^2^ in size [12], 12 of which were randomly allocated to receive deployments of *w*Mel-infected *Ae. aegypti* and 12 left untreated for the duration of the trial, but which received *w*Mel releases 6 months after the completion of the trial. Where possible, geographical borders such as roads, rivers, or non-residential areas were used as cluster boundaries to slow the dispersal of mosquitoes between clusters. The cluster boundaries used to define *w*Mel release areas were not aligned with the administrative (kelurahan) boundaries used for the purpose of routine disease notification, as illustrated in Figure 1. The study protocol and detailed description of the AWED trial are published [11].

### w*Mel deployment and monitoring*

As described previously [12], *w*Mel-infected *Ae.aegypti* mosquitoes were released in the 12 intervention clusters between March and December 2017. Entomological monitoring was performed by weekly collection of adult mosquitoes using a network of 348 BG Sentinel traps throughout the trial site. *w*Mel prevalence was calculated for each kelurahan as the proportion of screened *Ae. aegypti* mosquitoes in each kelurahan that tested positive for *w*Mel. As part of the Program’s commitment to the community, *w*Mel releases into previously untreated (control) areas was undertaken between October 2020 and January 2021. Monitoring of *w*Mel in Yogyakarta City was suspended between April and July 2020 due to the COVID-19 pandemic. There was one round of monitoring in August 2020 and some monitoring between October and December 2020 in 23 kelurahans where releases were ongoing. There has been no monitoring since the completion of *w*Mel releases in February 2021, except for one round of monitoring in 14 kelurahans in November 2021.

### Dengue surveillance system

Under the existing system for routine notification of dengue cases in Yogyakarta City, hospitals report cases diagnosed clinically as Dengue Hemorrhagic Fever (DHF; ICD-10 code A91; International Classification of Diseases 10th revision) to the Yogyakarta District Health Office. These case reports are not consistently accompanied by supportive laboratory testing. We collated data from January 2006 to May 2022 from this reporting system, aggregated by month and patients’ kelurahan of residence. Data on hospitalised dengue fever (DF) cases (ICD-10 code A90) were also obtained for January 2017–May 2022, as DF notification only began in 2017.

### Insecticide fogging for vector control

The Yogyakarta District Health Office undertakes focal spraying of insecticide (cypermethrin in 2016–2018 and malathion in 2019–2020) around the residence of notified dengue cases (hospitalised DHF cases only in 2016; DHF and DF from 2017 onwards), subject to resource availability and local transmission risk. Line-listed information on insecticide fogging for vector control, including fogging date and location, is maintained by the Yogyakarta District Health Office and was obtained retrospectively from January 2016 to August 2020 and aggregated by month and local *w*Mel status. Data on the annual cost of insecticide spraying for vector control was also obtained from the District Health Office. Insecticide fogging was paused from September 2020 to December 2021 due to the COVID-19 pandemic. As fogging activity had only partially resumed during the first half of 2022, we did not include 2022 data in our analysis.

### Statistical analysis

The impact of *w*Mel deployment on DHF and DF case notifications was evaluated using an interrupted time series (ITS) analysis of monthly case notifications by kelurahan, before and after *w*Mel releases. A notified case’s *w*Mel exposure status was determined by the wMel-treatment status of their kelurahan of residence in the month of case notification. The boundaries of the 35 kelurahans within the AWED trial site do not all align with the boundaries of the 24 AWED clusters, with 19 kelurahans straddling parts of both intervention and untreated clusters. Rules were defined to classify the *w*Mel exposure status of each kelurahan through time. Kelurahans were defined as ‘untreated’ prior to receiving any *w*Mel releases; ‘partially treated’ when releases had commenced in any part of the kelurahan, or if *w*Mel contamination had occurred (defined as kelurahan-level *w*Mel prevalence >50% for two monthly monitoring events within a 6-month rolling window and >50% of the BG traps in the kelurahan had detected *w*Mel-positive *Ae. aegypti* during those monitoring events); and ‘fully treated’ once *w*Mel releases had been completed in all parts of the kelurahan. All kelurahans were considered fully treated by February 2021 following the end of *w*Mel releases in all of Yogyakarta City. An alternative *w*Mel exposure definition used quintiles of kelurahan-level *w*Mel prevalence, calculated as a 3-month rolling average, as a predictor of dengue incidence. Here kelurahans can move across *w*Mel quintiles from month to month and thus each *w*Mel quintile in a given month may represent a different set of kelurahans. Mixed effects negative binomial regression was used to model the monthly count of DHF and DF case notifications in each kelurahan as function of *w*Mel treatment status (fully, partially or untreated in the primary analysis, and by quintile of *w*Mel prevalence in the secondary analysis), with an offset for population size, calendar month as a covariate to allow for seasonality, and with kelurahan modelled as a random effect.

A sensitivity analysis was performed excluding data after April 2020 when the COVID-19 pandemic and related restrictions may have resulted in hesitancy to attend healthcare facilities and changes to the diagnosis and reporting of dengue cases to the surveillance system during this period. An additional sensitivity analysis attempted to control for the potential confounding effects of secular trends in dengue transmission by restricting the data to the period when there were contemporaneous comparisons between untreated, partially treated and fully treated areas: from the month when *w*Mel releases began in the first AWED trial cluster (March 2017) to the month prior to the commencement of *w*Mel releases in the last kelurahan (September 2020), after which there were no longer any untreated areas in the city.

Geocoordinates of insecticide fogging applications were available, so these fogging events could be classified using the AWED trial cluster boundaries as occurring in intervention vs untreated clusters. Negative binomial regression was used to compare the number of insecticide fogging events in *w*Mel-treated clusters compared to untreated clusters, separately for the pre-intervention and post-intervention period. The post-intervention period was defined for each intervention cluster as beginning one month after the completion of releases in that cluster, and for the untreated clusters as beginning one month after completion of releases in the last intervention cluster.

## Results

The two-stage implementation of *Wolbachia* releases, first to areas randomly allocated to the intervention arm of the AWED trial and then to the untreated control areas after the trial, resulted in the 35 kelurahans in the trial area contributing a median of 136 months (range 134–177) to the ‘untreated’ *w*Mel status, 44 months (range 3–47) to the ‘partially treated’ status, and 17 months (range 16–56) to the ‘fully treated’ status between January 2006 and May 2022.

**Figure 2.**
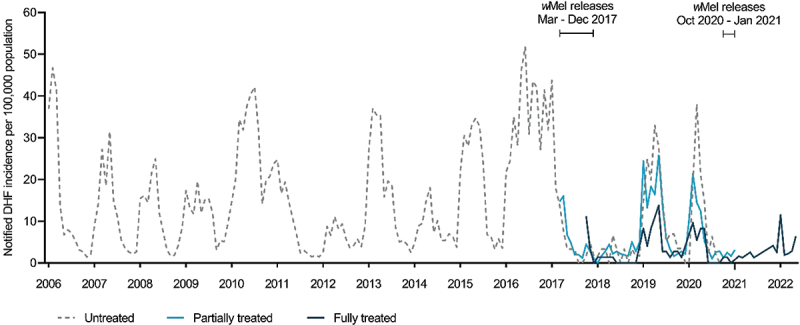
Incidence of notified dengue hemorrhagic fever in untreated, partially treated and fully treated kelurahans. Kelurahans were defined as ‘untreated’ prior to the commencement of *w*mel releases, ‘partially treated’ when releases have commenced in any part of the kelurahan or wMel contamination has occurred (kelurahan-level *w*mel frequency was >50% for two monthly monitoring events within a 6-month rolling window and >50% of the BG traps in the kelurahan have detected *w*Mel-positive *Ae. aegypti* during those monitoring events), and ‘fully treated’ once *w*mel releases were completed in all parts of the kelurahan.

Among the 8,362 DHF cases notified from the trial area during this same period, 7,603 were notified from untreated kelurahans (181.5 per 100,000 person-years), 537 from partially treated kelurahans (71.6 per 100,000 person-years), and 222 from fully treated kelurahans (33.3 per 100,000 person-years) (Figure 2), which is equivalent to a crude incidence rate ratio (IRR) of 0.18 (95% CI 0.16, 0.21) for fully treated vs untreated kelurahan-months and 0.39 (95% CI 0.36, 0.43) for partially treated vs untreated kelurahan-months. Among 783 DF cases notified between January 2017 (when DF became notifiable) and May 2022, 227 were notified from untreated kelurahans (80.9 per 100,000 person-years), 387 from partially treated kelurahans (51.6 per 100,000 person-years), and 169 from fully treated kelurahans (25.4 per 100,000 person-years), which is equivalent to a crude incidence rate ratio (IRR) of 0.31 (95% CI 0.26, 0.38) for fully treated vs untreated kelurahan-months and 0.64 (95% CI 0.54, 0.75) for partially treated vs untreated kelurahan-months.

**Figure 3.**
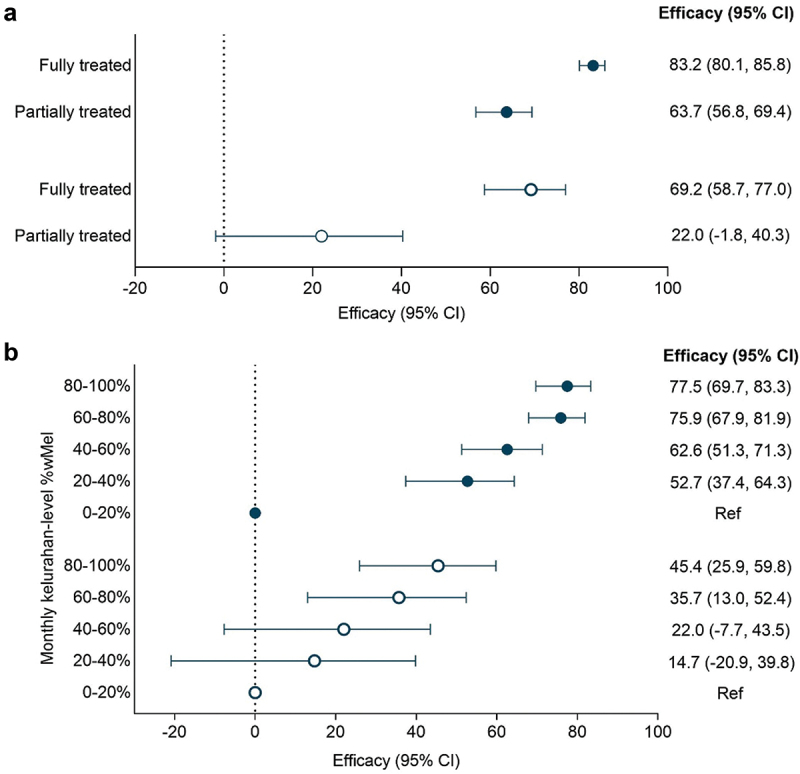
Efficacy of the *w*mel *Wolbachia* intervention against incidence of notified dengue hemorrhagic fever (closed circles) and dengue fever (open circles) by (a) *w*mel treatment status and (b) quintile of *w*mel. Point estimates (circles) and 95% confidence intervals (CI) (horizontal bars) from controlled interrupted time series analysis of monthly dengue case notifications to the Yogyakarta District Health Office. Efficacy was defined as 1-IRR (incidence rate ratio).

In the interrupted time series analysis, which adjusts for seasonal trends and between-kelurahan variability, the incidence of notified DHF was 83% lower in fully treated kelurahan-months (IRR 0.17 [95% CI 0.14, 0.20]) and 64% lower in partially treated kelurahan-months (IRR 0.36 [95% CI 0.31, 0.43]) compared to untreated kelurahan-months (Figure 3(a)). The incidence of notified DF was 69% lower in fully treated kelurahan-months (IRR 0.31 [95% CI 0.23, 0.41]) and 22% lower in partially treated kelurahan-months (IRR 0.78 [95% CI 0.60, 1.02]) compared to untreated kelurahan-months (Figure 3(a)).

**Figure 4.**
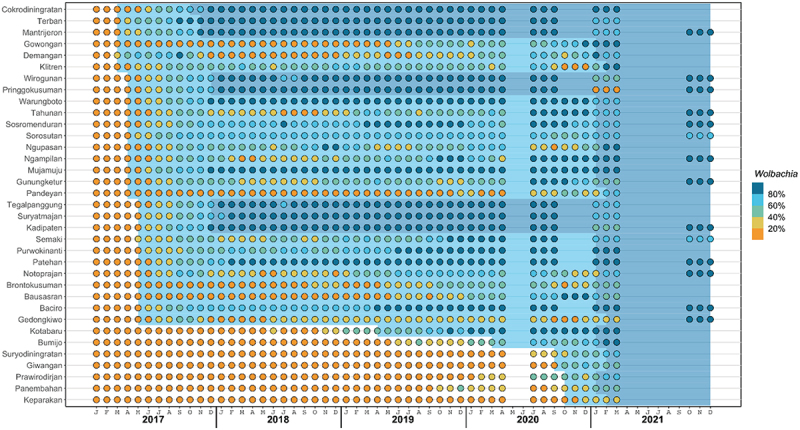
*W*mel introgression by kelurahan, 2017 – 2021. Circles indicate the 3-month moving average *w*mel infection prevalence in local *Aedes aegypti* mosquito populations categorised into quintiles, in each of 35 kelurahans (vertical axis) between January 2017 and December 2021 (horizontal axis). Light blue and dark blue background shading indicate the period during which each kelurahan is considered ‘partially treated’ and ‘fully treated’, respectively (see Methods for definitions).

*w*Mel monitoring was suspended between April – July 2020 and then ceased in 21/35 kelurahans after quarter 1 of 2021 (Figure 4), therefore the analysis using time-varying quintiles of kelurahan-level *w*Mel prevalence was performed using data to March 2021 only. The intervention effect displayed a dose–response relationship with quintiles of *w*Mel exposure (Figure 3(b)). DHF incidence was 78% lower in kelurahan-months with 80–100% *w*Mel (IRR 0.23 [95% CI 0.18, 0.30]) compared to kelurahan-months with 0–20% *w*Mel, and significant reductions in DHF incidence were also observed at 60–80% (IRR 0.24 [95% CI 0.18, 0.32]), 40–60% (IRR 0.37 [95% CI 0.29, 0.49] and 20–40% (IRR 0.47 [95% CI 0.36, 0.63]) *w*Mel prevalence. Only a marginal increase in intervention effect was observed at 80–100% *w*Mel (78% efficacy) compared to 60–80% *w*Mel (76% efficacy). A similar trend was observed when using the endpoint of notified DF, though the magnitude of the effect sizes were smaller and non-significant for the 40–60% and 20–40% *w*Mel quintiles (Figure 3(b)).

There is a possibility that reporting practices for many notifiable diseases, including dengue, were altered by the response to the COVID-19 pandemic, particularly during the height of the pandemic. Fear of being infected with COVID-19 may have led to avoidance of healthcare facilities for non-emergency conditions, and pressure on the healthcare system may have led to less reporting of dengue cases to the routine surveillance system, which together could result in an artificial decrease in notified dengue cases during this period that is unrelated to the *Wolbachia* intervention. To account for this possibility, a sensitivity analysis was performed where data from May 2020 onwards was excluded. The intervention effect estimated from this analysis was of slightly lower magnitude but consistent with that from the main analysis (Figure S1). A second sensitivity analysis restricted the dataset to the time during which there was contemporaneous comparison between areas that were untreated, partially treated and fully treated. This aimed to reduce the potential confounding effects of seasonality and interannual fluctuations in dengue incidence that can arise in before-and-after analyses. The intervention effect estimated in this sensitivity analysis was again lower than that from the primary analysis, though still clearly showed a reduction in dengue incidence in fully treated areas and in kelurahan-months with the highest *w*Mel prevalence (Figure S2).

**Figure 5.**
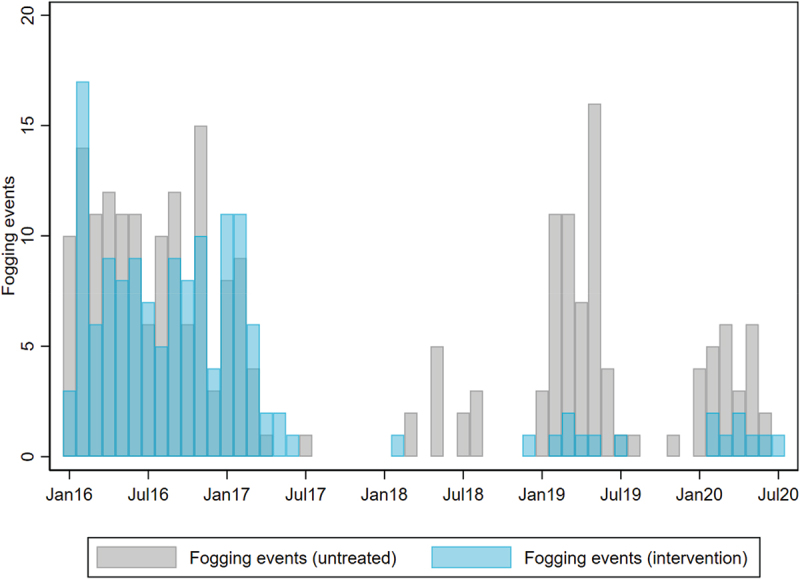
Insecticide fogging for vector control in *w*Mel-treated and untreated areas of Yogyakarta City by month. Focal spraying of insecticide (cypermethrin in 2016–2018 and malathion in 2019–2020) around the residence of notified dengue cases is done by the Yogyakarta District Health Office. Fogging events were aggregated by month and *w*Mel-exposure status for comparison between *w*Mel-treated and untreated areas.

At baseline (2016–2017), insecticide fogging activity occurred at similar frequencies in areas that were later randomised to receive *w*Mel deployments or to serve as untreated control areas in the AWED trial: median [interquartile range] of 7 [3–9] fogging events per month in treated areas vs 9 [3–11] in untreated areas (IRR 0.89 [95% CI 0.60, 1.32]) (Figure 5). After the completion of randomised *w*Mel releases into intervention areas, fogging activity occurred 83% less frequently in treated areas than in untreated areas (median [IQR] of 1 [0-1] events per month vs 3 [1–6]; IRR 0.17 [95% CI 0.10, 0.30]) (Figure 5). The annual cost spent on insecticide fogging in Yogyakarta City was reduced by 39.6% from USD 79,914 in 2016–2017 (prior to *w*Mel deployments into the AWED trial intervention areas) to USD 44,592 in 2018–2019 (after *w*Mel establishment in the intervention areas).

## Discussion

We show here that the establishment of *w*Mel *Wolbachia* in the local *Ae. aegypti* mosquito population significantly reduced the incidence of hospitalised dengue cases notified to the routine disease surveillance system in Yogyakarta and also the number of episodes of insecticide fogging in the community. These findings, from a predefined secondary analysis of a cluster randomised trial of *Wolbachia* for dengue control (the AWED trial; [12]), demonstrate the utility of routinely available public health data for evaluating the public health impact of *Wolbachia* deployments and the potential for large-scale *Wolbachia* deployments to dramatically reduce insecticide use and resourcing for routine *Aedes* control activities.

In the primary analysis of the AWED trial [12], the incidence of virologically confirmed dengue cases was reduced by 77% (95%CI 65%, 85%) and hospitalised dengue cases by 86% (95%CI 66%, 94%) in *w*Mel-treated areas of Yogyakarta city, between January 2018 and March 2020. The present study found a slightly higher reduction in notified dengue incidence of 83% (95% CI 80%, 86%) in *w*Mel-fully treated areas compared to untreated areas. The two analyses have several notable differences. The endpoint used in the AWED trial was virologically confirmed dengue cases detected among febrile patients presenting to outpatient clinics, while the current study used hospitalised DHF cases notified to the routine disease surveillance system. The AWED trial compared dengue incidence in 12 clusters randomised to receive *w*Mel deployments vs 12 clusters randomised to no intervention. In the present study, the discordance between the geographical boundaries used to define the randomised release of *w*Mel mosquitoes in the AWED trial (i.e. cluster boundaries) and the administrative boundaries used to report notified cases (i.e. kelurahan boundaries) meant that the *w*Mel status of dengue cases’ residential address could not be so cleanly defined, with 19 of the 35 kelurahans straddling both intervention and untreated clusters. *w*Mel exposure was thus assigned in the present analysis as ‘untreated’ prior to *w*Mel being deployed in any part of the kelurahan, ‘partially treated’ when any part of the kelurahan had received *w*Mel deployment (or contamination), and ‘fully treated’ only once *w*Mel deployment had finished in all parts of the kelurahan. Comparison of ‘fully treated’ vs ‘untreated’ kelurahan-months in this study should most closely resemble, though is not identical to, the comparison of intervention vs untreated clusters in the intention-to-treat analysis of the AWED trial. One advantage of the AWED trial design was the inclusion of test-negative controls to reduce residual confounding due to healthcare-seeking behaviours. Finally, the AWED trial compared two parallel study arms (intervention vs untreated) while the *w*Mel exposure classification in the current study was more similar to a non-randomised stepped wedge design with each kelurahan moving from ‘untreated’ to ‘partially treated’ to ‘fully treated’ at different times. The AWED trial intention-to-treat analysis and the secondary analysis described here both aimed to determine the efficacy of the *w*Mel intervention in reducing dengue incidence in Yogyakarta City, and despite using different endpoints, exposure definitions and analysis methods, they produced similar estimates of intervention effect, contributing to a growing body of evidence demonstrating large reductions in dengue incidence in areas where *w*Mel was deployed [7,8,10,12]. Importantly, the results of the present study show that at 60–80% wMel prevalence in the local *Ae. aegypti* population, the intervention effect was very similar to that at 80–100% *w*Mel prevalence. This supports previous findings from Brazil that *Wolbachia* deployments can significantly reduce dengue incidence even with imperfect levels of introgression [9,25].

In Indonesia, hospitals report dengue cases to the disease surveillance system on the basis of an ICD-10 code designated usually at the time of discharge from hospital. Coding is based on the clinical diagnosis, with or without confirmatory laboratory tests, such as NS1 antigen detection or nucleic acid tests for dengue virus RNA. By being hospital-based, the surveillance system underestimates true case incidence (imperfect sensitivity). By relying on clinical diagnosis for reporting, the surveillance system lacks specificity, as a variable proportion of notified cases may be a febrile illness of other aetiology (i.e. false-positive reports). These limitations make routine dengue case notifications an imperfect but pragmatic endpoint for measuring the effectiveness of a public health intervention like *Wolbachia*. The lower efficacy observed for hospitalised DF than for DHF in the present study is likely explained in large part by the lower specificity of a DF clinical diagnosis compared to DHF, meaning that a greater proportion of notified DF cases than DHF cases are likely to be misdiagnosed febrile illnesses of other aetiologies.

The findings of the current study support the use of interrupted time series analysis of routine dengue notifications data for assessing the public health impact of *w*Mel deployments under programmatic conditions, acknowledging that the original intervention randomisation may have significantly reduced bias and, as such, the current study is not purely observational. Attention needs to be paid to ensuring the boundaries used for *w*Mel releases and monitoring are aligned with the spatial units used for routine disease reporting wherever possible. Entomological monitoring should be granular enough to detect meaningful heterogeneities in *w*Mel establishment which can then inform a non-binary classification of area-level *w*Mel exposure status in the evaluation of public health impact. Case definitions for dengue notification are primarily clinically defined in most endemic countries, with some exceptions including Singapore and New Caledonia, where the majority of cases have laboratory confirmation [26,27]. Surveillance systems also vary widely in whether reporting includes ambulatory vs hospitalised cases and private vs public facilities. Nonetheless, as long as these notification settings are reasonably consistent across time (and between intervention areas and untreated control areas, where used), a valid estimate of the step change in dengue incidence following *w*Mel deployments can be made from dengue case notification time series data. These quasi-experimental methods have been applied successfully to measure the public health impact of *w*Mel *Wolbachia* releases in northern Australia [8,10]; Niteroi, Brazil [9]; and in previous releases on the periphery of Yogyakarta, Indonesia, prior to commencing the cluster randomised trial [7].

By substantially reducing the incidence of dengue, the *Wolbachia* deployments in Yogyakarta also significantly reduced the frequency of reactive applications of insecticide by environmental health teams around the homes of notified dengue cases, which was in turn associated with a 40% reduction in vector control spending in the city as a whole. For decades, only two chemical classes of insecticide (pyrethroids and organophosphates) have been used for the control of arbovirus vectors [28], leading to widespread resistance to these insecticides in mosquito populations in Latin America [29,30] and, more variably, in Southeast Asia [2,31]. Achieving a sustained reduction in insecticide use has the benefit of reducing the selective pressure that drives the evolution and spread of insecticide resistance in Aedes mosquitoes.

## Conclusions

We show that the efficacy of *Wolbachia* mosquito deployments for reducing dengue incidence that was demonstrated previously in a cluster randomised trial in Yogyakarta, Indonesia, was also measurable from routine surveillance data. City-wide *Wolbachia* deployments have now been completed throughout Yogyakarta city, as well as in urban centres in Latin America, northern Australia and the Pacific. In 2021 the first National Strategic Plan for Dengue Control in Indonesia (2021–2025) was launched [32], which identified *Wolbachia* as an evidence-based innovation for dengue control in Indonesia. Ongoing monitoring of dengue incidence and *Wolbachia* prevalence in Yogyakarta will provide additional evidence of the real-world effectiveness and durability of large-scale *Wolbachia* implementation. Our findings suggest that in addition to the direct public health benefits of this biological control tool, a secondary benefit of reduced insecticide use may help to preserve the efficacy of the available insecticides that will continue to play a role in an integrated approach to Aedes-borne disease control.

## Supplementary Material

Supplemental Material

## Paper context

A cluster randomised trial of *Wolbachia*-infected mosquito releases previously demonstrated the efficacy of this innovative method for dengue control in Yogyakarta, Indonesia. In this predefined secondary analysis of that trial, we show that the efficacy of *Wolbachia* was measurable also from time-series analysis of routine dengue surveillance data and that the intervention substantially reduced the frequency of insecticide fogging by public health teams. This highlights the broader benefits of applying *Wolbachia* for dengue control.
