# Supplementary material for: Impact of randomised wmel Wolbachia deployments on notified dengue cases and insecticide fogging for dengue control in Yogyakarta City
Source: Glob Health Action. 2023 Jan 26;16(1):2166650. doi: 10.1080/16549716.2023.2166650 (PMC9894080; doi:10.1080/16549716.2023.2166650)
Supplement: Supplemental Material [file ZGHA_A_2166650_SM6843.docx]

**SUPPLEMENTARY MATERIAL**

**
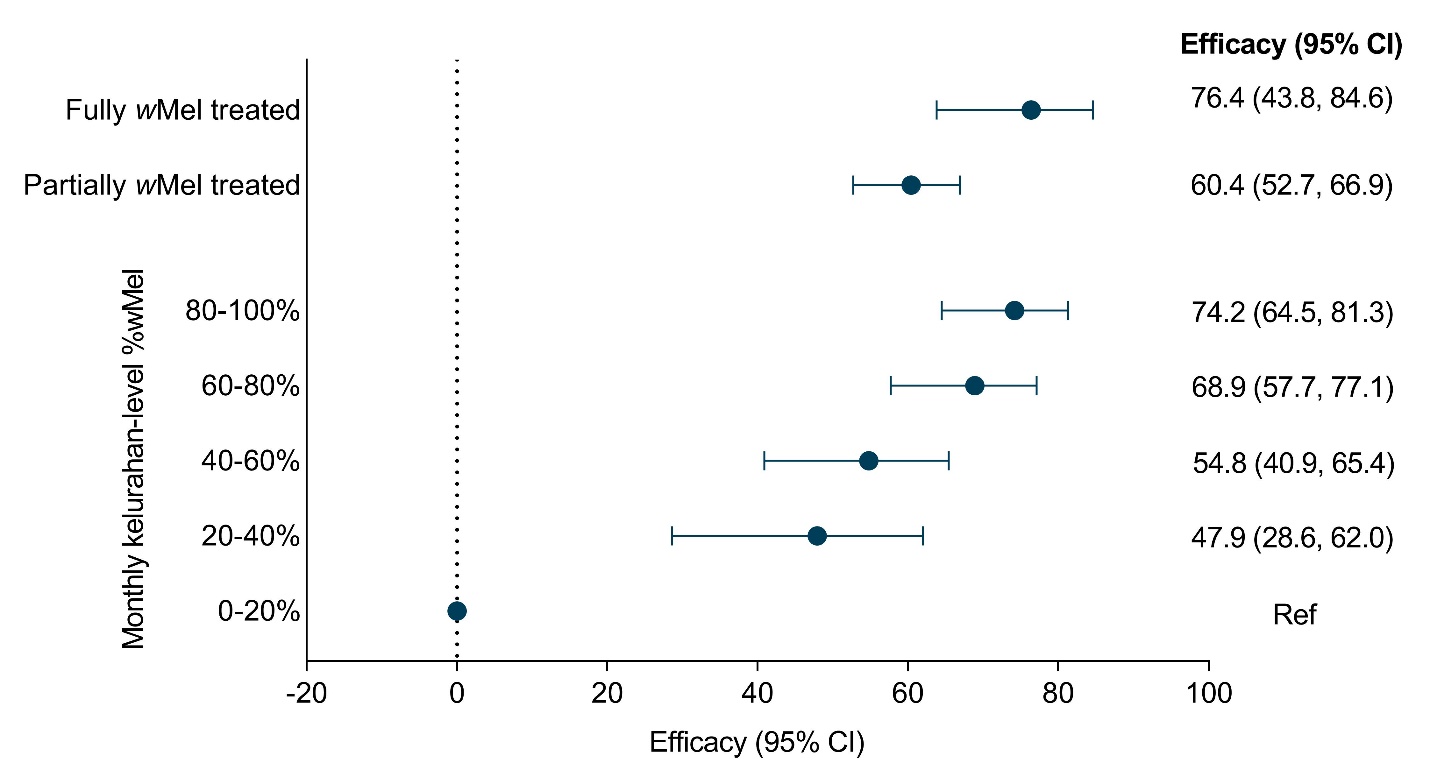
**

**Figure S1. Efficacy of the *w*Mel *Wolbachia* intervention against incidence of notified dengue hemorrhagic fever by *w*Mel treatment status (fully or partially treated vs untreated) and by quintile of *w*Mel: sensitivity analysis excluding the period potentially affected by COVID-19 pandemic restrictions.** Point estimates (circles) and 95% confidence intervals (CI) (horizontal bars) from controlled interrupted time series analysis of monthly dengue case notifications to the Yogyakarta District Health Office between January 2006 – April 2020. Efficacy was defined as 1-IRR (incidence rate ratio).

**
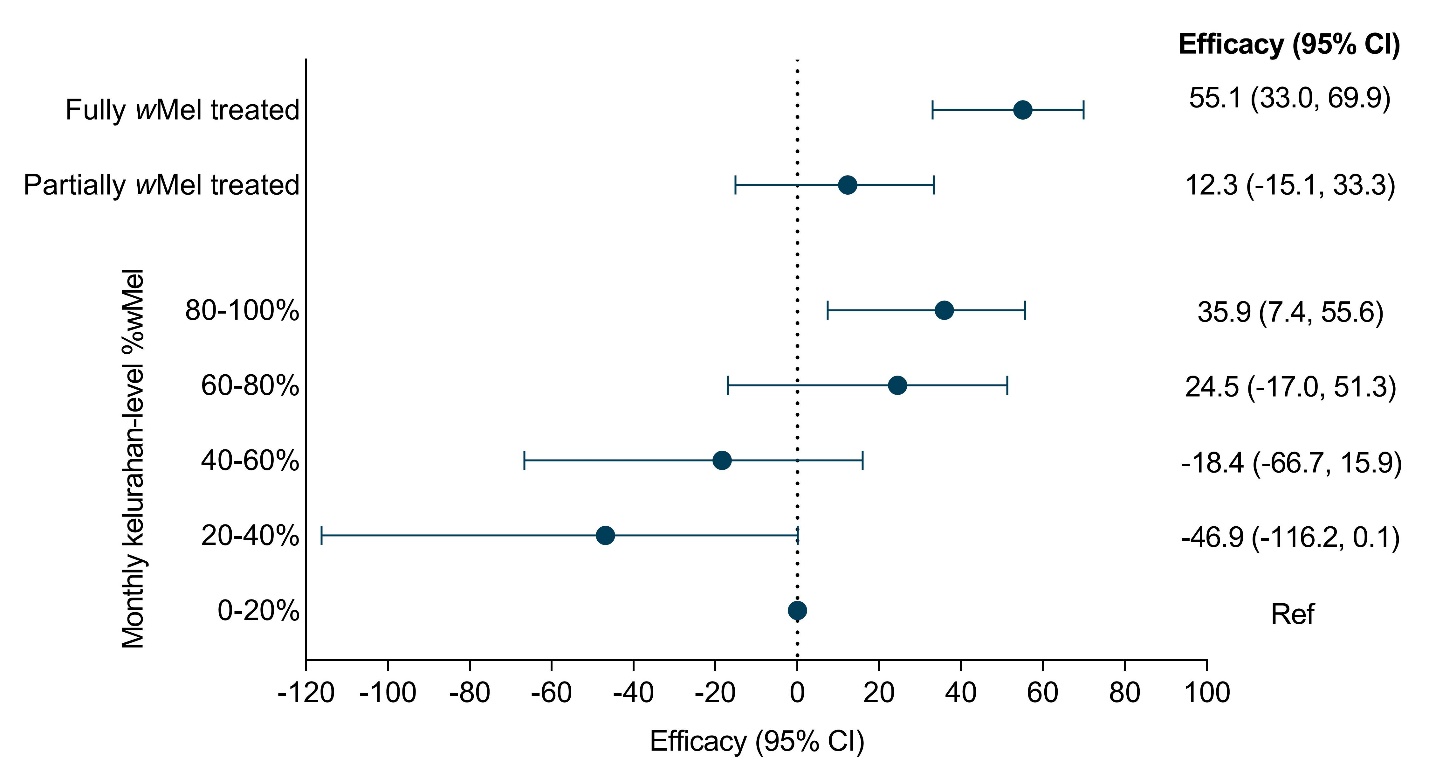
**

**Figure S2. Efficacy of the *w*Mel *Wolbachia* intervention against incidence of notified dengue hemorrhagic fever by *w*Mel treatment status (fully or partially treated vs untreated) and by quintile of *w*Mel: sensitivity analysis restricted to the period with contemporaneous untreated, partially treated and fully treated areas.** Point estimates (circles) and 95% confidence intervals (CI) (horizontal bars) from controlled interrupted time series analysis of monthly dengue case notifications to the Yogyakarta District Health Office between March 2017 – September 2020. Efficacy was defined as 1-IRR (incidence rate ratio).
